# Case Report: Anesthesia for Cesarean Section in Parturients With Chronic Renal Failure Requiring Hemodialysis: Case Reports and Literature Review

**DOI:** 10.3389/fsurg.2022.848496

**Published:** 2022-06-15

**Authors:** Ting Zhang, Xianwei Xiong, Yiling Jiang, Huan Chen, Juying Jin

**Affiliations:** Department of Anesthesiology, The First Affiliated Hospital of Chongqing Medical University, Chongqing, China

**Keywords:** Chronic renal failure, cesarean section, anesthesia, hemodialysis, uremia

## Abstract

Pregnancies are rare in women with chronic renal failure requiring hemodialysis. Although a chance of successful pregnancy and delivery in hemodialysis women has increased over the years, it is still very low, and with high maternal and fetal mortality and morbidity rate compared to normal population. And cesarean section is usually used for delivery. The first case was a 32-year-old Chinese woman with chronic kidney disease stage V undergoing maintenance hemodialysis for six years. The second case was also a 32-year-old patient with a 6-year history of hemodialysis for chronic glomerulonephritis. And due to a history of atrial septal defect and hypertension she received intensive hemodialysis during pregnancy. Both cases were scheduled for cesarean delivery under epidural anesthesia. To help develop reasonable anesthetic methods and management objectives for such patients, we described our anesthetic management and performed a literature search on published cases of cesarean section with chronic renal failure undergoing hemodialysis.

## Introduction

Patients with chronic kidney disease (CKD) are less able to make the renal adaptations needed for a healthy pregnancy. In the past, any degree of renal impairment was considered incompatible with pregnancy and those patients were usually advised to terminal the pregnancy (1). In fact, since a study in 1971 reported the first case of a 35-year-old end-stage renal failure woman on hemodialysis (HD) who achieved a full-term pregnancy, the further cases followed (2). The effect of pregnancy on maternal kidney disease may manifest as a loss of renal function. CKD, even when mild, can lead to the high risk of adverse pregnancy outcomes, including preeclampsia, preterm delivery, and small-for-gestational age infants or neonatal intensive care unit (NICU) admission, and these risks increase with the severity of the underlying renal dysfunction, degree of proteinuria, as well as the frequent coexistence of hypertension (3). A study reported that compared to patients without kidney disease, those with kidney disease had 52% increased odds of preterm delivery and 33% increased odds of delivery via cesarean section (4). Compared with babies born to parturients without kidney disease, babies born to parturients with kidney disease had 71% increased odds of admission to neonatal intensive care unit (NICU) or infant death and the chance of low birth weight has tripled (4).

Anesthesia for such patients entails many risks, such as changes increased gastric acid secretion, cardiovascular instability, in drug metabolism and platelet dysfunction (5). For such patients, anesthesia management is very important, but few literatures mentioned it at present. In this paper we obtained the written informed consent of the patients and shared our experience of epidural anesthesia in parturients with chronic renal failure (CRF) on HD.

## Case Report

### Case 1

A 32-year-old woman with 3 gravida and one para (G3P1) was scheduled for cesarean delivery at 36 weeks and two days’ gestation. She had delivered a live baby 12 years before due to umbilical cord around the neck. She weighed 63.5 kg and was 150 cm tall. She had a 17-year history of chronic glomerulonephritis and has been on HD three times per week after progressing to CKD stage V six years before. The family history of this patient was unremarkable. During pregnancy, the patient’s renal function was stable without complications such as anemia, hypertension, and edema. She was admitted to the hospital for cesarean section due to obstetrical B-ultrasound indicating severe fetal growth restriction (FGR). She received heparin-free HD 13 h before operation. Her laboratory data during her admission is summarized in the Table 1.

**Table 1:** Preoperative, postoperative and discharge laboratory values.

		Preoperative		Postoperative (the day after the operation)		Discharge	
		Case 1	Case 2	Case 1	Case 2	Case 1	Case 2
Blood	RBC (×10^12^/L)	3.33	3.59	2.95	3.46	3.07	3.47
	Hemoglobin (g/dL)	11.7	10.0	10.1	10.0	10.6	9.8
	Haematocrit (%)	36.1	33.5	31.4	32.4	32.4	31.5
	Platelet count (×10^9^/L)	161	339	146	352	174	324
Coagulation tests	INR	0.92	1.06	–	–	–	–
	PT (seconds)	10.5	12.2	–	–	–	–
	APTT (seconds)	27.4	28	–	–	–	–
	Fibrinogen (g/L)	6.94	5.22	–	–	–	–
	D-dimer (mg/L)	2.22	1.86	–	–	–	–
Renal function and electrolytes	Urea (mmol/L)	18.7	34.2	18.9	–	11.6	39.1
	Creatinine (µmol/L)	978	927	888	–	666	1011
	K (mmol/L)	4.7	4.7	4.1	5.6	4.1	5.3
	Na (mmol/L)	139	138	–	136	135	132
	Ca (mmol/L)	2.23	2.31	2.15	–	2.07	2.19
	Cl (mmol/L)	105	103	–	99	99	93
	Total protein (g/L)	62	70	–	–	51	–
	Albumin (g/L)	35	37	–	–	27	–
Arterial blood gases	PH	7.45	7.50	–	–	–	–
	PaCO_2_ (mmHg)	34	33	–	–	–	–
	PaO_2_ (mmHg)	106	90	–	–	–	–
	$\mathrm{HC}O_{3}^{-}$ (mmol/L)	24.2	25.7	–	–	–	–
	Glucose (mmol/L)	4.8	4.9	–	–	–	–
TEG	R (min)	7.3	5.4	–	–	–	–
	K (min)	1.4	0.8	–	–	–	–
	Angle (deg)	69.5	80.2	–	–	–	–
	MA (mm)	72.5	73.3	–	–	–	–

*ACT, activated clotting time of whole blood; INR, international normalized ratio; PT, prothrombin time; APTT, activated partial thromboplastin time; TEG, thrombelastography.*

Noninvasive blood pressure (NIBP:100/62 mmHg), electrocardiography (ECG), heart rate (HR:95/ min), and SpO_2_ (98%) were monitored after entering the operating room and invasive arterial catheterization was performed from the right radial artery under local anesthesia (because an arteriovenous fistula was being used for HD on the left side). After checking the normal thrombelastography (TEG) and activated clotting time of whole blood (ACT), and their international normalized ratio (INR), prothrombin time (PT), activated partial thromboplastin time (APTT) and platelet count are normal, considering that there was no contraindication of epidural anesthesia, we decided to perform cesarean section under epidural anesthesia.

**Figure 1:**
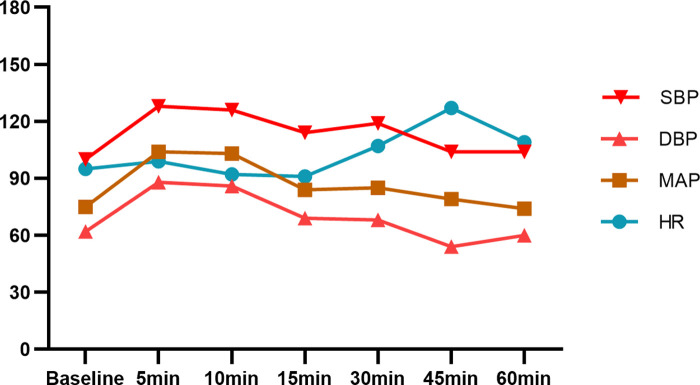
BP and HR of case 1 after epidural anesthesia. SBP, systolic blood pressure; DBP, diastolic blood pressure; MAP, mean artery pressure; HR, hearte rate.

Epidural anesthesia with 5 mL test dose of 2% lidocaine was successfully performed at the L1-2 intervertebral space in the right-lateral position. After 5 min, there was no general spinal anesthesia and local anesthetic poisoning, and 2% lidocaine 7 mL was added. Epidural anesthesia level having reached the T8 to S dermatomes, the operation was started. A 1,770 g baby with an Apgar score of 10 in one minute and 10 in five minutes was delivered 12 min after the beginning of surgery. Invasive blood pressure and HR were stable during the surgery and Figure 1 shows the values. No headache, nausea or vomiting were present during surgery. The operation lasted for 50 min, with 1,100 mL intraoperative crystalloid fluid, 200 mL bleeding, and no urine.

After the operation, the epidural catheter was removed after measuring the anesthesia level (T10-S). No bleeding was observed at the puncture point, and the patient was sent back to the ward after the patient controlled intravenous analgesia (PCIA) was placed.

### Case 2

Another 32-year-old G4P1 woman had a 6-year history of dialysis for chronic glomerulonephritis. Her weight was 62 kg and her height was 158 cm. The parturient had a history of atrial septal defect and hypertension and received intensive HD (5 / week) during pregnancy. The patient regularly took nifedipine controlled-release tablets 30 mg QD for hypertension. During pregnancy, her blood pressure was poorly controlled, fluctuating between 150–190/70–100 mmHg, and it was deemed necessary to add labetalol. The patient has anemia and disorders of electrolytes like most patients with end-stage renal disease. She used erythropoietin (EPO) regularly for a long time and increased the dose during pregnancy. Preoperative investigations showed hemoglobin of 10 g/dL and normal serum potassium. During pregnancy, the woman did not have edema of both lower extremities. A decision to perform cesarean section was taken at 33 weeks and 5 days due to heart failure in the context of a viral infection. Her Brain Natriuretic Peptide (BNP) was over 35,000 ng/L. And the echocardiogram of this patient showed atrial septal defect, left atrium and left ventricle enlargement, pulmonary artery systolic pressure (PASP) 35 mmHg, and left ventricular ejection fraction (LVEF) 42%. Her last dialysis was performed 17 h before surgery using heparin-free HD.

**Figure 2:**
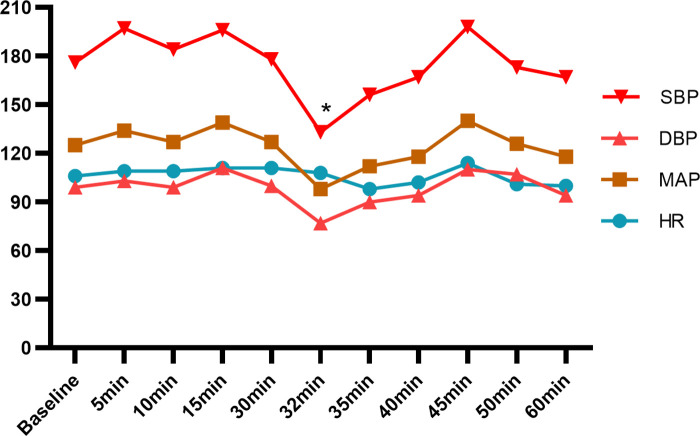
BP and HR of case 2 after epidural anesthesia. SBP, systolic blood pressure; DBP, diastolic blood pressure; MAP, mean artery pressure; HR, hearte rate. *intravenous injection of 50 µg phenylephrine.

In the operation room, monitoring included NIBP (176/99 mmHg), ECG, HR (106/min), and SpO_2_ (99%), but urine output was not monitored. Invasive arterial catheterization was performed and the TEG was normal. Epidural anesthesia was successfully given at the L2-3 intervertebral space using 4 mL 2% lidocaine in the right-lateral position. 11 mL 2% lidocaine was added after observation without general spinal anesthesia and local anesthetic poisoning. The operation began when the anesthesia level having reached the T8 to S dermatomes. After 7 min, a 1,885 g live baby was delivered (Apgar score: 9-9). Hypotension occurred after the baby was delivered, and we injected 50 µg phenylephrine intravenously and her BP and HR during the surgery are shown in Figure 2. The operation lasted for 42 min, with 600 mL intraoperative crystalloid fluid, 200 mL bleeding. Her cardiac and renal condition was not aggravated during the surgery.

After the surgery, no complications of anesthesia were observed, the epidural catheter was removed after measuring the anesthesia level (T10-S) and the patients was transferred to the cardiology intensive care unit (CCU) for further monitoring and returned to the ward the day after the operation. Both newborns were transferred to NICU.

Both mothers returned to heparin-free dialysis on the first postoperative day. During the following days, the two women were in stable condition, and recovered without any anesthesia-related complications such as epidural hematoma occurred headache, nausea or neurological deficit. They continued regular dialysis 3 times per week after they were discharged from the hospital. Table 1 shows their laboratory data for the day after the operation and the last data before discharged. After months of follow-up, their renal function has been stabilized by regular HD and their babies are growing up healthily. Table 2 shows the maternal and infant outcomes of the two cases.

**Table 2:** Maternal and infant outcomes.

	Discharge	HD frequency	Renal function	BP and medication	Anemia and medication	Infant outcome
Case 1	The third day after operation	3/week	stable	Normal /−	Slightly lower /−	survived
Case 2	One week after operation	3/week	stable	Slightly higher / nifedipine controlled-release tablets 30 mg QD	Slightly lower /EPO	survived

*HD, hemodialysis; BP, blood pressure; QD, quaque die; EPO, erythropoietin.*

## Discussion

Chronic renal failure (CRF) is the common outcome of the continuous progression of all kinds of CKD. Because of the damage of renal parenchyma, the basic renal function cannot be maintained. Pregnancy is uncommon in patients with end-stage renal failure. Uremia-associated hypothalamic-pituitary dysfunction and malnutrition usually result in amenorrhea and anovulation, and thus disturbances in menstruation and fertility are commonly encountered in women with CRF, leading to a low pregnancy rate in these patients (6). In addition, due to the teratogenic effect of drugs the fertility of patients with kidney disease is reduced (3, 7). However, the number of pregnant women treated with long-term HD has been increasing recently. Even with unwanted pregnancy, achieving successful pregnancy in such patients is challenging as adverse pregnancy outcome including preeclampsia, FGR, preterm delivery and accelerated loss of maternal renal function is significantly increased with higher degrees of renal impairment (3, 8, 9). In addition, anemia, vitamin D deficiency and chronic hypertension are comorbidities that are associated with adverse pregnancy outcome and can develop secondary to underlying CKD (9). CKD may affect placental perfusion and lead to placental insufficiency (7, 10). Additionally, fetuses with placental insufficiency habitually shunt blood away from nonessential organs, such as kidneys, to decrease urine output and thus amniotic fluid volume, leading to oligohydramnios and even fetal distress (7). It is important to control anemia because it is a significant cause of left ventricular hypertrophy, heart failure and angina. As women with CKD may have insufficient capacity for a gestational increase in EPO production, supplementation with synthetic EPO may be required (9). The hematocrit should be optimized before surgery. The correction of anemia also helps to improve platelet dysfunction associated with renal failure (11). Hypertension is the most common life-threatening problem in these patients. Controlling hypertension is an important part of CKD management during pregnancy and ideal blood pressure control should be optimized before pregnancy (3). The safety of antihypertensive drugs in pregnancy is based on historical, retrospective analyses and favours older classes of drugs, including the adrenergic receptor agonists labetalol and methyldopa and the calcium channel blocker nifedipine (9). Hyperkalemia is another important problem for patients with CRF. Hyperkalemia is usually induced by insufficient hemodialysis, acidosis, hypoxemia, and decreased body temperature. It is generally believed that preoperative fasting can reduce the level of potassium. However, a previous study found that the hormone response to fasting may transfer the potassium from the intracellular to the extracellular space (12). Therefore, close monitoring of electrolyte status during the perioperative period is necessary.

Due to presence of severe FGR and high induction rate for maternal indications like severe preeclampsia or worsening renal function, the incidence of cesarean section in pregnancy with CKD is higher (1). And the number of pregnant women with CRF requiring HD has increased recently. For patients undergoing cesarean section with HD, the choice of anesthetic methods and intraoperative management are very important. The primary task of the selection of anesthetic methods for such patients is to ensure the safety of the mother and the fetus, to minimize the adverse effects of pregnancy and kidney disease on mother and consequent effects on the fetus, and to avoid aggravating the renal failure of patients (13). Patients with CRF have a high risk of anesthesia, to help choose the appropriate anesthetic technique for such patients, a literature search of published case reports of pregnancy associated with CRF requiring HD was performed and listed in Table 3.

**Table 3:** Pregnancy cases in patients with CRF requiring HD in review.

Author	Year	Gestation week	HD frequency	HD history (month)	Fetal weight(g)	Apgar Score	Anesthetic technique
Kobayashi (14)	1981	38 wk and 6 d	5/week	48	1,320	5–7	epidural anesthesia
Azuma (15)	1996	38 wk	4–6/week	120	2,575	8–9	spinal anesthesia
Masako (5)	2000	32 wk and 3 d	5/week	192	NR	8–9	general anesthesia
Dhir (10)	2007	36 wk	7/week	144	1,729	6–8	epidural anesthesia
Zencirci (11)	2010	37 wk and 5 d	3/week	36	2,220	6–8	spinal anesthesia
Fernandes (16)	2011	NR	3/week	2	2,000	−9	spinal anesthesia
Cao (17)	2018	31 wk and 4 d	3/week	48	1,700	8–10	general anesthesia
Zhang	This case	36 wk and 2 d	3/week	72	1,770	10-10	epidural anesthesia
Zhang	This case	33 wk and 5 d	5/week	72	1,885	9-9	epidural anesthesia

*HD, hemodialysis; NR, not reported.*

Regional anesthesia and general anesthesia are both used for cesarean delivery and both have advantages and disadvantages. It is important to clarify what type of anesthesia is more suitable for such patients. CKD may affect both the pharmacokinetics and the pharmacodynamics of a drug. In general anesthesia, there is a possibility of altered drug clearance leading to accumulation of active metabolites which could be nephrotoxic (18). And general anesthesia may lead to decreased renal blood flow in such patients, thus affecting drug clearance rate, duration of action and efficacy intensity (19). In addition, decreased gastric motility and delayed gastric emptying in parturients may increase the risk of aspiration during general anesthesia (11, 19). When general anesthesia is performed on patients with CKD, it is necessary to consider the effects of an altered clearance, the production and accumulation of active metabolites, and the risk of aggravating pre-existing kidney disease on drug administration (13). In addition, these neonates often have intrauterine growth retardation, low birth weight and premature delivery are prone to the adverse effects of anesthetic agents (13). Due to the rapid and predictable effects of general anesthesia, the majority being performed for lack of time in the setting of an emergency delivery such as severe fetal distress, or in cases of failure or contraindications of intrathecal anesthesia (20).

In cesarean delivery, regional anesthesia using spinal or epidural anesthesia is a safe technique to minimize the risk of fetal depression and aspiration pneumonia in the mother (13). Compared with general anesthesia, it is possible for regional anesthesia to establish a connection between the mother and newborn immediately after delivery. However, abnormal coagulation function in patients with CRF and long-term HD treatment may increase the risk of intrathecal anesthesia bleeding. There is no absolute contraindication to regional blocks in women with CRF undergoing HD. Some literatures have pointed out that if anticoagulants have been used during HD, at least six hours should elapse before siting a regional block (11, 16, 21). It is wise to confirm that clotting is normal by checking the APTT, PT and TEG. Spinal anesthesia is a safe technique for such women. The dosage of local anesthetic agents which are used for spinal anesthesia has been reported to exert no influence upon the baby (5). However, most cases of spinal anesthesia experience hypotension followed by decreased uterine blood flow (5), which in severe cases can cause dizziness, nausea and vomiting.

Compared with general anesthesia and spinal anesthesia, epidural anesthesia can be used for cesarean section after labor analgesia failure and for postoperative analgesia after cesarean section. In our case, B-ultrasound of both parturients showed severe FGR, and one of the parturients was complicated with atrial septal defect. Compared with general anesthesia, epidural anesthesia can reduce venous return and alleviate the cardiac burden (22). In addition, epidural anesthesia is easier to maintain hemodynamic stability than spinal anesthesia (22). In order to avoid the effect of general anesthesia on maternal and fetal prognosis and unstable hemodynamic caused by spinal anesthesia, we chose epidural anesthesia. Previous literatures have pointed out that cesarean section under epidural anesthesia is safe for patients with heart disease (22, 23). However, before performing epidural anesthesia, attention must be paid to the coagulation function of CRF patients undergoing dialysis.

HD without heparin has long been used in maintenance dialysis patients with bleeding risk (24). In chronic HD patients with high risk of bleeding, heparin-free HD is now considered as a good option and recommended by anesthesiologists (25). In addition, thromboprophylaxis in these women was also important. The perioperative management for the two patients including thromboprophylactic agent administration was based on the collaboration of a multidisciplinary team (MDT), which was comprised of obstetrician, nephrologist, cardiologist, anesthesiologist and neonatologist. According to the suggestion of the experts in the MDT, the two patients had a relatively low risk for venous thromboembolism (VTE) during pregnancy and after surgery. Furthermore, coagulation tests and ultrasonography revealed no signs of VTE. As a result, thromboprophylactic agent was not administered during perioperative period. In our case, heparin-free dialysis was performed one day before the operation. After the preoperative examination confirmed that the blood coagulation function and internal environment were normal, the cesarean section was successfully completed under epidural anesthesia. No complications of epidural anesthesia occurred in the postoperative follow-up, and their renal function were stable. It is suggested that with careful and effective monitoring preoperatively and intraoperatively, epidural anesthesia can be safely performed for cesarean section in patients undergoing heparin-free HD.

## Conclusion

Renal disease, either preexisting or occurring during pregnancy may impair maternal and fetal health. Although the number of pregnant women undergoing HD is rare, the risk of anesthesia is extremely high and there are multiple anesthetic considerations in such parturients. The choice of anesthesia for such patients should be made on an individual basis. Based on our cases, we consider that epidural anesthesia is safe to the patients with CRF requiring HD if there are no coagulation abnormalities. And heparin-free HD is considered as a good option for such patients to reduce the risk of bleeding during the perioperative period. However, the number of cases in this study is small, and a large number of studies are required to determine the safety of epidural anesthesia for such patients. In addition, since the condition of two patients remained stable, the central venous pressure was not monitored during the operation. if possible, it is necessary to strengthen intraoperative hemodynamic monitoring. In short, by perfecting relevant examinations before operation, correcting patient's internal environment and blood coagulation function and closely monitoring during the operation, the patient can still achieve a good outcome.

## Data Availability

The original contributions presented in the study are included in the article/Supplementary Material, further inquiries can be directed to the corresponding author/s.
